# An Analysis of the Alleged Skeletal Remains of Carin Göring

**DOI:** 10.1371/journal.pone.0044366

**Published:** 2012-12-19

**Authors:** Anna Kjellström, Hanna Edlund, Maria Lembring, Viktoria Ahlgren, Marie Allen

**Affiliations:** 1 Osteoarchaeological Research Laboratory, Department of Archaeology and Classical Studies, Stockholm University, Stockholm, Sweden; 2 Department of Immunology, Genetics and Pathology, Rudbeck Laboratory, Uppsala University, Uppsala, Sweden; University of Illinois at Champaign-Urbana, United States of America

## Abstract

In 1991, treasure hunters found skeletal remains in an area close to the destroyed country residence of former Nazi leader Hermann Göring in northeastern Berlin. The remains, which were believed to belong to Carin Göring, who was buried at the site, were examined to determine whether it was possible to make a positive identification. The anthropological analysis showed that the remains come from an adult woman. The DNA analysis of several bone elements showed female sex, and a reference sample from Carin's son revealed mtDNA sequences identical to the remains. The profile has one nucleotide difference from the Cambridge reference sequence (rCRS), the common variant 263G. A database search resulted in a frequency of this mtDNA sequence of about 10% out of more than 7,000 European haplotypes. The mtDNA sequence found in the ulna, the cranium and the reference sample is, thus, very common among Europeans. Therefore, nuclear DNA analysis was attempted. The remains as well as a sample from Carin's son were successfully analysed for the three nuclear markers TH01, D7S820 and D8S1179. The nuclear DNA analysis of the two samples revealed one shared allele for each of the three markers, supporting a mother and son relationship. This genetic information together with anthropological and historical files provides an additional piece of circumstantial evidence in our efforts to identify the remains of Carin Göring.

## Introduction

Born October 21, 1888 in Stockholm, Sweden, Carin was the daughter of Baron Carl Alexander Fock and Huldine Beamish. In 1910, she married Nils Gustav von Kantzow and three years later they had a son. The marriage has been described as unhappy, and when she and the decorated pilot Hermann Göring met in 1920 they fell in love. In 1923 she remarried and became Carin Göring [1] and the couple settled down in Germany. In November 1923, due to the “Beer Hall Putsch” where Göring played an important role, they had to leave Germany. However, the political situation in Germany was unstable and the coup makers were given amnesty in 1927, enabling the couple to return. Being the wife of one of the most central leaders within the growing National Socialist German Workers' Party (NSDAP) gave Carin high social status [2]. Adolf Hitler liked her, and she has been called the mascot of the Nazi party [3]. Carin suffered from heart problems and during her last years she was admitted on and off into different nursing homes. In October 1931, during a visit in Sweden, she died of heart failure and was buried in the family tomb at Lovön, on the island Ekerö outside Stockholm. Three years later Hermann moved the remains to his country residence, Carinhall, named after her, near Berlin. The funeral, worthy of a statesman, was a propaganda success, with all the most prominent Nazi leaders attending, including Hitler. The original coffin was placed in a coffin made of zinc and this in turn was placed in a tin coffin. The mausoleum at Carinhall played an important role in many Nazi meetings during the Second World War. In 1945, to hinder the Russians from getting hold of his belongings, Göring destroyed Carinhall. However, the mausoleum seems to have remained without too much damage. The history of the remains after this event is somewhat unclear. In 1951, according to the writer and journalist Björn Fontander, skeletal elements presumably belonging to Carin were discovered near Carinhall. The remains, described as “not more than a human torso” were handed over to a minister and transferred to the Swedish church in Berlin, cremated and buried in the family tomb in Stockholm [3]. Forty years later, in 1991, treasure hunters found a zinc coffin with skeletal remains in the Schorfheide Forest northeast of Berlin, again near the location of the destroyed Carinhall. The men used a video camera to document the excavation. The story has been published in an article in the (former East) German magazine *Super Illu* 1991 [4]. These remains were also handed over to the Swedish church in Berlin, which sent it to the Swedish National Board of Forensic Medicine for examination and individual identification.

In 2009, the skeletal elements were examined in detail at the Rudbeck Laboratory, Uppsala University. First an osteological investigation was performed followed by a DNA analysis for a possible identification of the individual. The remains were also compared to the video recording from 1991, which was kept together with the human remains. To deal with potential degradation of the DNA, mitochondrial DNA (mtDNA) is frequently used for DNA analysis of aged skeletal remains [5], [6], [7]. The cytoplasmic mtDNA exists in many more copies, compared to autosomal DNA, which is situated in the nucleus of human cells. Another feature of mtDNA is the strict maternal inheritance pattern resulting in maternal lineages. This is useful in relationship studies and provides a possibility of using a maternal relative as a source for reference material (e.g. Carin's son). For all these reasons, an initial analysis of mtDNA was performed on the putative remains of Carin Göring. Moreover, a molecular sex determination was performed. Finally, to increase the evidentiary value of the genetic information, analysis of nuclear markers was performed.

## Methods

### Osteological methods

The identification of the remains was an assignment from the Swedish National Board of Forensic Medicine. Since identification analysis is one of the clinical aims at the forensic departments, no ethical approval was requested from the regional ethics committee. Morphological features of the skull were used for sex assessments according to Buikstra and Ubelaker [8]. Metric data from the scapula, the clavicle, the humerus and the radius were used for sex assessment. For the glenoid cavity of the scapula, work by Stewart [9], was utilised, and a regression formula for caucasoid individuals was applied for the measurement of epicondylar breadth of the distal humerus as in France 1983 [10]. For both the clavicle and the radius, metric methods based on the Tennessee Data Bank from European and African Americans were used [10]. For age estimation ectocranial suture closure was used [11]. The stature estimation of the radius is based on the work of Trotter and Gleser [12].

### Contamination precautions

A DNA analysis of aged skeletal remains requires special safety precautions in order to avoid contamination by modern exogenous DNA. Therefore, a special clean-room facility, with HEPA-filtered air, positive pressure and LAF benches was used. To avoid contamination from the analysts, full body laboratory coats, facial masks, hair covers and disposable gloves were worn at all times. Separated pre and post polymerase chain reaction (PCR) laboratories were used, and each step of the analysis was performed by at least two different analysts. Furthermore, numerous negative controls were included in the extraction procedure, and PCR and all working areas as well as the equipment were regularly UV irradiated and cleaned with sodium hypochlorite (bleach). The genetic profiles of the staff handling the pre-PCR steps were known and were all compared with the obtained profile.

### DNA extraction of skeletal remains

An ulna bone and part of the cranium were selected for the DNA analysis. A total of two pieces (approximately 1 cm^3^ each) from the cranium and four pieces from the ulna were sampled using a Dremel drill. The bones were soaked in 6% commercial bleach (NaOCl) for 15 minutes followed by three washing steps in sterile H_2_O to remove exogenous contamination [13], [14]. For demineralisation of the bones, 2 ml of 0.5 M ethylene diamine tetra-acetic acid (EDTA) (pH 8) was added and the bone samples were incubated at 25°C for 52 h. Thereafter, 3 mg Proteinase K (20 mg/ml) was added and the samples were incubated at 65°C for 12 h under agitation at 300 rpm. The supernatant was transferred to a new 15-ml tube, and isolation of DNA was performed using the Wizard genomic purification kit (Promega Corporation) according to the manufacturer's protocol with minor modifications. The supernatant was mixed with 2 ml nuclei lysis solution followed by incubation for 3 h at room temperature. Thereafter, 1.3 ml protein precipitation was added followed by centrifugation for 5 minutes at 9000 rpm. The supernatant was divided into two 15-ml tubes and 99% isopropanol was added for precipitation of DNA in −20°C for 12 h. The precipitation was followed by centrifugation for 30 minutes at 9000 rpm, and the supernatant was then discarded. EtOH (70%) was added followed by centrifugation for 5 minutes at 9000 rpm. The supernatant was discarded and the pellets were dried for 4 h and then re-suspended in 400 µl rehydration solution. The DNA extracts were stored in −20°C until use.

### DNA extraction of paraffin-embedded tissue

As a reference in the identification analysis of Carin Göring's putative remains a formalin-fixed paraffin-embedded (FFPE) tissue sample from Carin Göring's son, Thomas Kantzow, was used. Thus, the maternal relationship could be investigated by comparing the mtDNA sequences as well as search for shared alleles of nuclear DNA (nDNA) markers between samples. DNA from the tissue was extracted by cleaning the tissue block and cutting a section of 5 mm^3^ tissue into smaller pieces. Extraction was performed in a 1.5-ml tube containing 150 µl extraction buffer composed of 0.61 g TrisBase, 0.5% TWEEN 20, 1 mM EDTA and H_2_O. The sample was incubated at 65°C for 6 h followed by addition of 50 µl extraction buffer and 0.2 mg Proteinase K, and the sample was incubated again at 65°C for 12 h. This was followed by deactivation of the proteinase at 95°C for 10 minutes and precipitation of DNA as described above.

### PCR for mtDNA analysis

The hypervariable regions I and II (HVI and HVII) in the control region of the mitochondrial genome are routinely sequenced in forensic genetics and ancient DNA analysis [15]. For PCR and sequence analysis the HVI primers 16128 and 16348 as well as the HVII F-45 and R-287 were used (Table 1). The resulting PCR fragments are 221 bp for the HVI region and 243 bp for HVII. To investigate the degree of degradation in the samples, the hypervariable region I was also amplified using three different primer pairs, generating short (221 bp), intermediate (440 bp) and long (616 bp) amplification products (Table 1). In order to counteract inhibitors, dilutions with water in 1∶10 and 1∶20 concentrations were prepared from the original extracts. Each PCR reaction contained 10 µl DNA extract (undiluted, 1∶10 or 1∶20) and 1× PCR Gold Buffer (Applied Biosystems), 0.2 mM dNTPs, 2.4 mM MgCl_2_ (Applied Biosystems), 10% Glycerol, 0.16 mg/ml BSA, 0.2 µM of each primer and 5 U AmpliTaqGold™ (Applied Biosystems) in a total volume of 30 µl. Amplification was performed in a GeneAmp PCR System 9700 instrument (Applied Biosystems) and the cycling conditions were 1 cycle of 10 minutes at 95°C, 40 cycles of 30 seconds at 95°C, 45 s at 60°C, 60 s at 72°C with a final extension step for 7 minutes at 72°C for all 4 targets.

**Table 1:** Primer sequences and cycling conditions used for amplification.

Name	5′ Primer sequence	DNA region	Fragment size
IFb-16128	GGTACCATAAATACTTGACCACCT	HVI	221 bp
IR-16348	GACTGTAATGTGCTATGTACGGTAAA		
IIFa-45	ATGCATTTGGTATTTTCGTCTG	HVII	243 bp
IIR-287	TTGTTATGATGTCTGTGTGGAAAG		
15971	TTAACTCCACCATTAGCACC	HVI	440 bp
16410	GAGGATGGTGGTCAAGGGAC		
15971	TTAACTCCACCATTAGCACC	HVI	616 bp
R17	CCC GTG AGT GGT TAA TAG GGT		
Amelogenin F	CCCTGGGCTCTGTAAAGAATAGT	Chr X and Y	106 bp (XX)
Amelogenin R	ACTAGAGCTTAAACTGGGAAGCTG		112 bp (XY)

### Sequencing of mtDNA

The PCR products were purified using two methods, the QIAquick® PCR purification kit (Qiagen, Hilden, Germany) and with Exonuclease I (ExoI) (Thermo scientific, Waltham, MA, USA) and FastAP thermosensitive Alkaline Phosphatase (Thermo scientific) in a mixture. Sanger dideoxy sequencing was performed using the ABI PRISM®Big Dye™ terminator Cycle Sequencing Ready Reaction kit v3.3 (Applied Biosystems). Sequencing reactions were run on an ABI Prism 3730 instrument (Applied Biosystems) and the sequencing data was analysed using the Sequencher 4.5 software package (Gene Codes Corporation, Ann Arbor, MI, USA). The obtained mtDNA sequences were compared to a reference sequence, the revised Cambridge reference sequence (rCRS), and deviations were reported as sequence differences to rCRS with Genbank accession number NC_012920 [16], [17]. The mtDNA database EMPOP (www.empop.org) was used to estimate the frequency of a particular mtDNA sequence. When comparing two mtDNA sequences, at least two differences between them are required for a conclusive exclusion [15].

### Sex determination

A DNA-based sex determination of the skeletal remains was performed, based on analysis of the amelogenin gene (AMEL). The gene is located both on the X chromosome (AMELX) and the male-specific Y chromosome (AMELY) and a common target for sex determination in forensic DNA analyses is a six bp deletion on the X chromosome [18]. The amelogenin region was amplified using 0.2 µM of each primer (Table 1), 10 µl DNA extract, 1×PCR Gold Buffer (Applied Biosystems), 0.2 mM dNTPs, 1.5 mM MgCl_2_ (Applied Biosystems), 10% Glycerol, 0.16 mg/ml BSA, and 5 U AmpliTaqGold™ (Applied Biosystems) in a total reaction volume of 30 µl. The cycling conditions were 10 minutes at 95°C, followed by 45 cycles of 30 seconds at 95°C, 45 seconds at 55°C, 60 seconds at 72°C, and a final extension step of 7 minutes at 72°C. The PCR products were sequenced using the Pyrosequencing technology, which is based on sequencing by synthesis, where incorporation of nucleotides results in generation of light [19]. Purification of templates and generation of single stranded products were performed according to the SQA template preparation protocol using the PSQ 96 Sample Preparation Kit and Streptavidin Sepharose™ High Performance beads (Quiagen, Hilden, Germany). The PSQ™96 SQA reagent kit was used for sequencing, and the reactions were run on a PSQ 96MA instrument using the SQA analysis in the PSQ 96MA (version 2.1) software. The generated light is proportional to the number of incorporated nucleotides, and the DNA sequence can be read based on the appearance of a peak and the height of the signal in a pyrogram. A homozygous pattern illustrates the presence of a six bp deletion on both chromosomes, evincing a female. A heterozygous result indicates the presence of one X chromosome and one Y-chromosome for a male individual.

### Analysis of nDNA

In order to increase the evidentiary value, an nDNA analysis was performed using a small set of Short Tandem Repeat (STR) markers used in forensic genetics. The best performing markers in an assay previously developed for pyrosequencing analysis, TPOX, TH01, D5S818, D7S820 and D8S1179 were included in the analysis [20]. Amplification of DNA was performed in 30 µl reactions containing 0.2 mM of each dNTP, 2.5 mM MgCl_2_, 1×PCR Taq Gold buffer (Applied Biosystems), 10% Glycerol, 0.16 mg/ml BSA, 5 U AmpliTaq Gold® DNA Polymerase (Applied Biosystems), 0.2 µM of each primer and 10 µl of DNA. Thermal cycling (Gene Amp PCR system 9700, Applied Biosystems) was performed with an initial hot start at 95°C for 10 minutes followed by 45 cycles at 95°C for 30 s, 53°C for 30 s, and 72°C for 30 s. An annealing temperature of 60°C was used for TH01. The final extension was carried out at 72°C for 7 minutes. Template preparation and pyrosequencing was performed as described by manufacturer and the samples were run on a PyroMark Q24 platform, version 2.0.6 Build 2.0 (Qiagen)

## Results

### The general appearance

**Figure 1:**
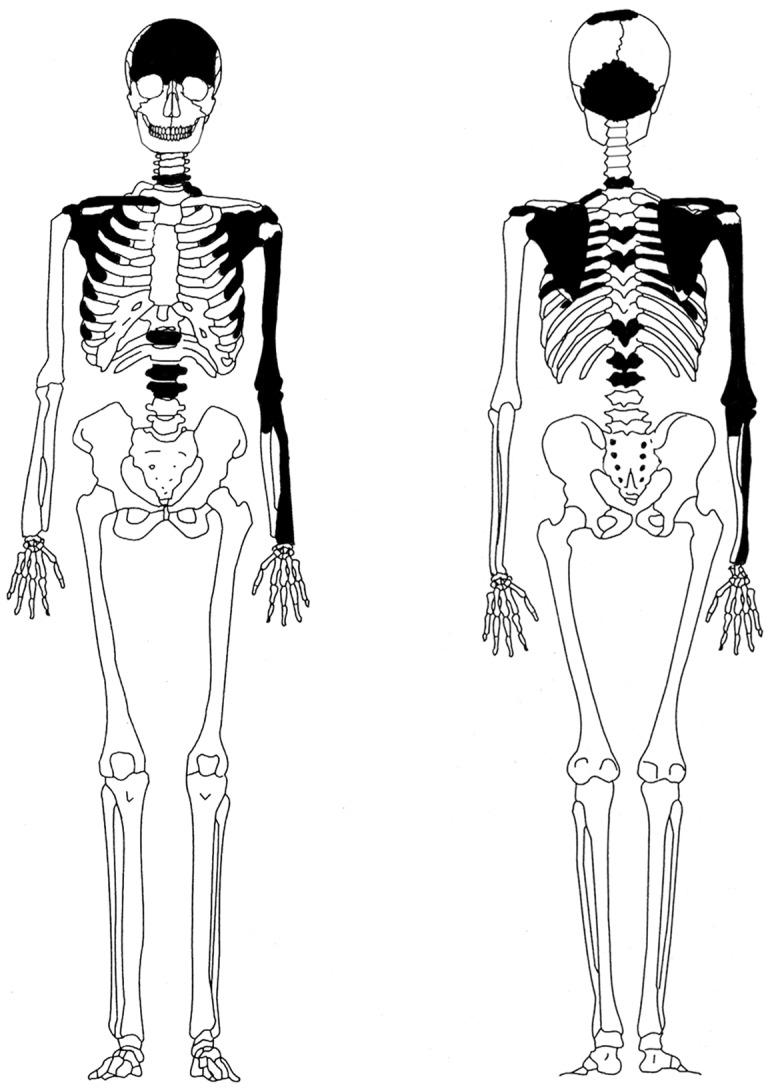
Schedule showing preserved elements (in black).

In total 26 bones from both the cranium and the upper postcranial body were received from the Sweden National Board of Forensic Medicine (Figure 1 and Table 2). The elements showed different signs of postmortem trauma (e.g., loss of the proximal diaphysis of the humerus) and some surface erosion but were in general firm in character. Based on the facts that all bones were of the same colour, the same elements but from different sides were equivalent in size and shape, and some elements showed a trim articulation – it is likely that they belonged to the same individual. Even though the video from the treasure hunters is of poor quality, similarities are seen between the bones being discovered and excavated in the film with the physical remains analysed in this study. For instance, in the film a humerus, which is broken proximally, is shown, a complete radius is displayed and close-ups are taken of a single frontal bone and an occipital bone. These bone elements demonstrate a close resemblance in character, colour and fragmentation to the analysed remains.

**Table 2:** Bone preservation and measurements.

Element	Preservation (% of complete bone)	Measurements (mm)
Frontal bone	100%	
Occipital bone		
Parietal bone (left and right)	20% (area around Bregma)	
Right clavicle	100%	
Left clavicle	100%	GL 135.0
		ant-post 13.13
		inf-sup 8.27
Right scapula	100%	Cav. glen. 36.5
Left scapula	100%	Cav. glen. 33.5
Left radius	100%	GL 244.0
		ant-post 10.75
		med-lat 15.72
Left ulna	c. 25%	
Left humerus	c. 75%	Max Mid 20.01
		Min Mid 19.90
		GdB 57.5
Cervical vertebra x1	100%	
Thoracic vertebrae x3	100%	
Lumbar vertebrae x2	one 100%, one fragmented (40%)	

(GL = greatest length, ant-post = anterior-posterior diameter, inf-sup = inferior-superior diameter, med-lat = medial-lateral diameter, Max Mid = maximal diameter at midshaft, Min Mid = minimal diameter at midshaft, GdB = greatest distal breadth).

### The anthropological analysis

The sex characteristic features (including *the supra-orbital margin*, *the supra-orbital ridge* and *glabella* on the frontal bone together with *the nuchal crest* of the occipital bone) were gracile implying that the skull bones are derived from a woman. The measurements of the clavicle, radius and left scapula and the distal epicondylar breadth of the humerus also suggest that the individual was a woman. The result of the measurement of the right scapula places the individual in the interval between female and male. All elements are fully developed and the sternal end of the clavicle is fused. The parts of the coronal and sagittal sutures, which were still preserved, are ectoranially closing and in stage 1 according to the stages presented by Meindl and Lovejoy [11]. Except for some porosity at the sternal end of the clavicle, no degenerative processes of the joint surfaces are visible. The remains were clearly from an adult. The poor bone representation makes it difficult to present a detailed age estimate. If the regression equation based on white females is used for the radius, the woman is indicated to have been c.170.6±4.24 centimetres.

**Figure 2:**
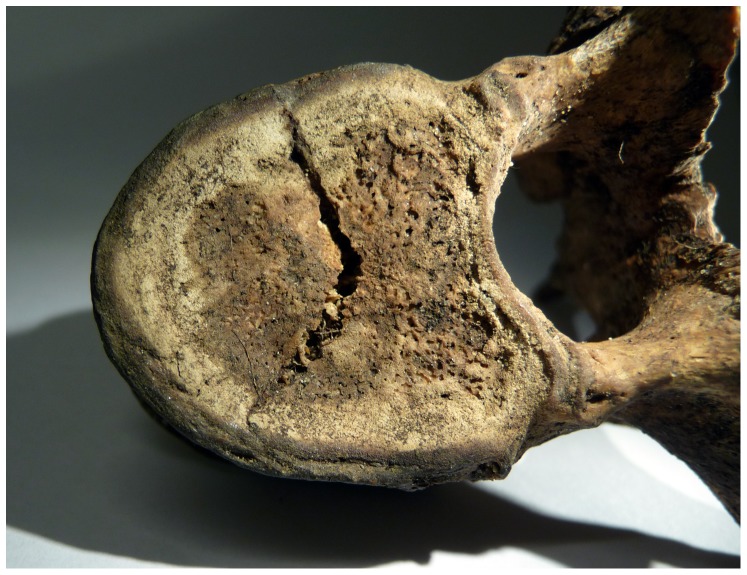
Photograph showing thoracic vertebra trauma.

**Figure 3:**
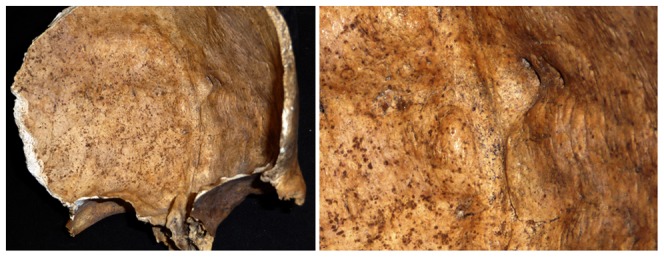
Photograph showing irregular bony outgrowths on both sides of the frontal crest.

One of the thoracic vertebrae (T6-T8?) shows sign of a V-shaped trauma on the superior surface of the body (Figure 2). The fracture radiates from the centre and terminates at the vertebral rim. The posterior fracture edge is pressed into the body and below the anterior fracture edge. The character of the cancellous bone renders the edges irregular, making it difficult to distinguish whether it was a perimortem or postmortem fracture. Centrally on the endocranial surface close to the frontal crest of the frontal bone a thickened area with one large (10×5 mm) and two minor (5×5 mm) dense, protruding, round nodules with discrete margins was observed (Figure 3). The largest nodule, located on the left side of the frontal crest, protruded approximately 4 mm. The surface of the thickened area is smooth, but the endocranial tabula exhibits clusters of swollen billows superior to the nodules, foremost close to the right side of the coronal suture. The outer table exhibits no pathological changes.

### Analysis of mtDNA

A total of six DNA extracts were obtained, four from the ulna and two from the cranium. The degree of degradation in the samples was estimated by amplification of mtDNA with primer pairs generating short (221 bp), intermediate (440 bp) and long (616 bp) amplification products (Table 1). In total, ten PCR reactions were set up for each fragment size. The long HVI fragment failed to yield positive PCR reactions, while the short fragment revealed positive amplification (with different intensity in gel electrophoresis) for all PCR trials. The intermediate sized fragment of 440 bp was amplified in approximately half of the attempts. Thus, the samples show an inverse relation between success rate and fragment length, a typical behaviour for aged and degraded material.

**Figure 4:**
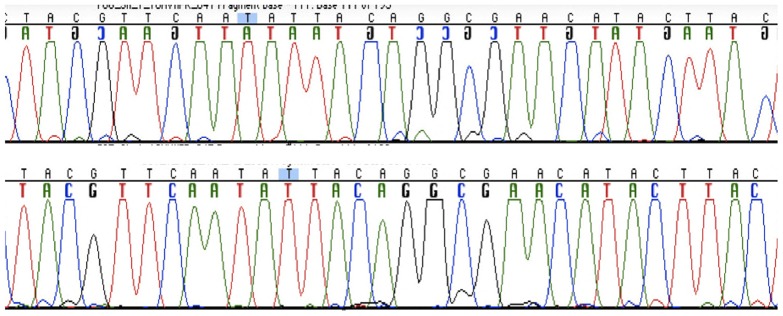
Sequencing chromatograms showing identical sequences of a part of HVII from the skull sample (upper chromatogram) and the formalin-fixed paraffin-embedded tissue sample used as reference.

For sequence analysis of mtDNA, a total of 36 PCR reactions were set up using the primer pairs generating 221 and 243 bp, of the HVI and HVII regions, respectively. Each DNA extract was amplified undiluted and at dilutions of 1∶10 and 1∶20. Approximately one third of the amplifications revealed products and were successfully amplified and sequenced. PCR products were obtained from all six extracts, although the ulna samples amplified more successfully. While the samples from the remains could be amplified in longer fragments, the paraffin embedded tissue yielded only the short fragment. The sequence analysis revealed identical results between the parts from the cranium, the ulna and the reference sample between nucleotides 16153 and 16322 for HVI and 73 and 263 for HVII. One single sequence was obtained from the six extracts and the negative amplification controls were negative. The majority of the sequences were of high quality, although a few miscoding lesions were seen (Figure 4). The HVI sequences were identical to rCRS, while all samples have a single difference compared to rCRS in HVII position 263. This common A263G substitution was also detected in the sample from Carin's son, and the sequence differs in several positions from the analysts. When a complete match between two samples is obtained, as in this case, the rarity of the profile in the population will determine the evidentiary value. MtDNA sequences are more or less common in various populations and for estimation of the frequency: the mitochondrial population database (EMPOP) (www.empop.org; Version: 2.1, Release 7) was used. The database contains mitochondrial DNA profiles from 17 104 individuals and the search for the profile (A263G) revealed that this mtDNA sequence is found with a frequency of 6.4% in all populations or in 10.3% among Europeans (total number of 7585 individuals).

### Analysis of nDNA

**Figure 5:**
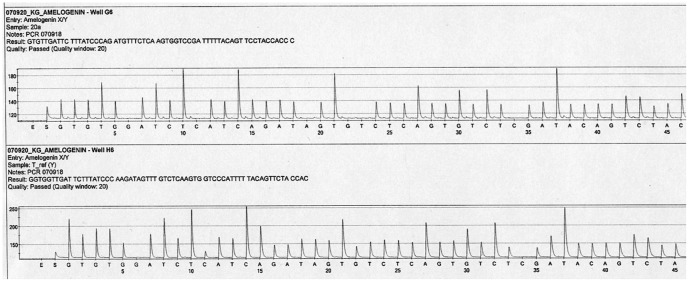
Pyrosequencing results for a part of the amelogenin gene for sex determination. The upper pyrogram (skull sample) indicates a female individual, which can be seen by the different sequence pattern from dispensation 19 to 23 due to the six-bp deletion female individuals have in this part of the amelogenin gene. The lower pyrogram (the reference material) shows a male individual.

The sex determination by pyrosequencing analysis was consistent with the anthropological data and revealed that the cranium and ulna originate from a female individual. In addition, the paraffin-embedded tissue sample from Carin Göring's son was sex determined and confirmed to originate from a male individual (Figure 5). In general, analysis of mtDNA has low discrimination capacity, and the haplotype in this particular case is very common. Therefore, an nDNA analysis was attempted to further improve the evidentiary value. A first test, using the AmpliSTR Identifiler (STR) Kit from Applied Biosystems, failed to yield results from the remains. Further analysis was performed using the STR markers TPOX, TH01, D5S818, D7S820 and D8S1179, which were amplified in short targets to facilitate analysis of aged and degraded samples [20]. Several products for the TH01, D7S820 and D8S1179 markers could be obtained from both samples for further pyrosequencing analysis and genotyping. The TPOX and D5S81 markers did not amplify in either the remains or the reference sample and were therefore excluded in further analysis. The results for the amplified and sequenced markers are summarised in Table 3. The two samples share an allele for each of the three markers supporting a mother and son relationship. Statistical evaluation for the STR markers resulted in a combined likelihood ratio (LR) of 26.9 in favour of the hypothesis that the remains are from the mother of the reference individual. When the mtDNA data is added, the LR is increased to 277 (Table 3).

**Table 3:** STR genotypes and mtDNA determined in the remains and the reference sample.

Marker	Remains	Frequency*	Tissue/Tomas	Frequency*	LR**
TH01	8/9	0,08/0,13	8/9	0,08/0,13	4,98
D7S820	9/9	0,21	8/9	0,10/0,21	2,37
D8S1179	13/13	0,22	12/13	0,01/0,22	2,28
mtDNA	A263G	0,103	A263G	0,103	10,3

Frequency* - allele frequencies determined in the Swedish population (Divne et al. 2010).

LR** - Likelihood ratios.

## Discussion

An anthropological analysis was made of the bone elements for a thorough primary documentation of the skeletal preservation and of macroscopic bone changes. Moreover, mitochondrial DNA analysis, a molecular sex determination, and nuclear DNA typing were performed, which all support the notion that the bones discovered in the Schorfheide Forest in 1991 are the remains of Carin Göring.

The bone changes of the frontal bone demonstrate a close resemblance with hyperostosis frontalis interna (HFI). HFI is a disease, which results in irregular nodular thickening of the internal table of the frontal bone. The etiology of the disease is not established, but it seems to be connected to obesity and diabetes among women after menopause [21]. The changes are most often observed in women over 60 years of age but the disease has been diagnosed in women younger than 40 years. Furthermore, studies have showed that mild cases can be missed and that it may be a misconception that HFI is an “old-age phenomenon” [22]. The nodules are in line with type A characters of HFI described by Hershkovitz and colleagues [22]. The most frequently mentioned differential diagnoses include other hyperostoses of the skull such as Paget's disease, fibrous dysplasia, acromegaly and benign sclerotic solitary bone mass, e.g. osteomas [22], [23]. However, in Paget's disease a poorly mineralised osteoid matrix results in a “cotton wool” appearance with both lytic and sclerotic changes. The irregular thickening often involves the entire skull, including both the ectocranial and the endocranial surfaces. The skull is affected in 10–25% of cases with monostotic fibrous dysplasia [24]. The disorder may lead to a replacement of bone by proliferated fibrous tissue in localised areas. Acromegaly causes an enlargement of the frontal sinuses and involves both the endocranial and the ectocranial surfaces [25]. Osteomas are most often found on the ectocranial surface and in most cases are a single lesion [26]. Based on the morphology, localisation and bilateral distribution of the nodules together with the age and sex data, the individual might have suffered from HFI.

Apart from comments about her beauty, she has been described as tall [1]. Since the mean stature for women in Sweden today (2003) is 165.5 cemeteries [27] and it was even less in the 1920s, it is likely that the remains with the estimated height of c. 170 centimetres based on the radius, is from a tall woman of the time. According to a friend of Carin's, she was said to have suffered not only from heart problems but also from asthma [3]; in 1923 she was said to have had pneumonia [2] and in 1925 tuberculosis [2], [28]. During the lawsuit regarding the custody of her son, her former husband submitted a doctor's certificate saying that Carin suffered from epilepsy [1], [2]. The osseous material cannot confirm (or refute) these notions. Asthma and epilepsy do not primarily involve the skeleton. The visceral surface of the ribs, which may be affected by pleural infections [29], does not exhibit any bone alterations.

The initial genetic analysis of the remains involved a sex determination analysis based on analysis of a sequence pattern difference between females and males, due to a six basepair (bp) deletion on the X chromosome. The results of this analysis showed that the remains are of female origin and the reference sample from her son, Thomas, of male origin. The remains and the reference sample were further examined by mtDNA analysis to investigate maternal relationship. Since mtDNA is maternally inherited, a mother and her offspring share an identical mtDNA sequence. The two samples display an identical mtDNA sequence suggesting a maternal relationship. However, due to degradation of the DNA, only a part of the hypervariable regions could be amplified and sequenced from the FFPE sample. Approximately 180 bp each of the HVI and HVII control regions were successfully analysed. Overall, the FFPE sample provided the largest challenge in the analysis, but the fact that the remains were degraded made these difficult to analyse in larger fragments as well. The particular mtDNA sequence obtained in this case is one of the most common types seen among Caucasians [30]. As a consequence, 10% of Europeans share identical DNA data with the bone samples and the reference sample according to the EMPOP database (www.empop.org).

Aged skeletal remains are often highly degraded, and different environmental factors can affect the bones negatively. In particular, bones that have been buried are subjected to microorganisms, fungi and humic acid that will accelerate degradation of the DNA. However, as approximately 400 bp fragments could be amplified from the ulna sample, the remains appear to be relatively well preserved, perhaps because they were not buried in soil for a very long period. The indication of degradation makes it less likely that the products are a result of modern contamination. Moreover, the cranial bone seems to be somewhat more degraded than the ulna bone, as only a few of the intermediate sized fragments could be amplified. In addition, when an organism dies, intracellular enzymes such as nucleases break the DNA molecule into short fragments. All these factors complicate a molecular analysis of aged skeletal remains. For these reasons, nuclear DNA analysis is most successful if short targets are used [31]. In this study we were able to successfully use a subset of STR markers that were analysed by pyrosequencing technology [20]. Out of five tested markers, three yielded PCR products and interpretable genotypes from both the putative remains of Carin and the sample from Thomas. For all three markers, alleles were shared in support of a mother son relationship. Thus, we have both mitochondrial and nuclear DNA data supporting that the remains are those of Carin Göring.

## Conclusions

The results of the anthropological analysis show that the remains found in 1991, identified as the ones depicted in a contemporary video, come from an adult woman. The DNA analysis revealed that the remains are from a female. Further analysis of the ulna, cranium and a reference sample from Carin's son revealed identical mtDNA sequences. The sequence displays one difference to the rCRS (A263G) and an mtDNA database search resulted in a frequency of about 10% among 7585 European haplotypes for this particular profile. The mtDNA sequence found in the ulna, cranium and reference sample is thus very common among Europeans. Finally, a nuclear DNA analysis of the remains and the son supports a mother and son relationship, adding a higher evidentiary value to the identification. Thus, the osteological and genetic information obtained in this study, together with additional anthropological and historical data, provides several pieces of evidence in the identification of the remains of the former Nazi leader Hermann Göring's wife, Carin Göring.
